# Large‐scale DNA methylation profiling of urological cancers identifies shared and cancer‐specific methylation signatures, and reveals differentially activated pathways for therapeutic targeting

**DOI:** 10.1002/ctm2.70488

**Published:** 2025-10-13

**Authors:** Vera Constâncio, João Lobo, Antonio Gomez, João Ramalho‐Carvalho, Isa Carneiro, Rui Freitas, Manuel Esteller, Rui Henrique, Carmen Jerónimo

**Affiliations:** ^1^ Cancer Biology & Epigenetics Group IPO Porto Research Center (CI‐IPOP) Portuguese Oncology Institute of Porto (IPO Porto) Porto Portugal; ^2^ Porto Comprehensive Cancer Center Raquel Seruca (Porto.CCC) Porto Portugal; ^3^ Doctoral Program in Biomedical Sciences ICBAS ‐ School of Medicine & Biomedical Sciences, University of Porto Porto Portugal; ^4^ Department of Pathology Portuguese Oncology Institute of Porto (IPO Porto) Porto Portugal; ^5^ Department of Pathology and Molecular Immunology ICBAS ‐ School of Medicine & Biomedical Sciences, University of Porto Porto Portugal; ^6^ Department of Biosciences Faculty of Sciences and Technology (FCT) University of Vic – Central University of Catalonia (UVic‐UCC) Barcelona Catalonia Spain; ^7^ Urology Clinic Portuguese Oncology Institute of Porto (IPO Porto) Porto Portugal; ^8^ Cancer Epigenetics Group Josep Carreras Leukemia Research Institute (IJC) Badalona Catalonia Spain; ^9^ Centro de Investigacion Biomedica en Red Cancer (CIBERONC) Madrid Spain; ^10^ Institucio Catalana de Recerca i Estudis Avançats (ICREA) Barcelona Catalonia Spain; ^11^ Physiological Sciences Department School of Medicine and Health Sciences University of Barcelona (UB) Barcelona Catalonia Spain

1

Dear Editor,

We report a comparative DNA methylation profiling study across prostate (PCa), bladder (BlCa), and kidney (KCa) cancers, uncovering both shared and cancer‐specific epigenetic traits underlying tumourigenesis. Our findings highlight distinct methylation signatures, with implications for early detection and the development of targeted therapies in urological malignancies.

Altogether, urological cancers represent the most common malignancies worldwide, with a 2.5‐fold increase in incidence since 1990, accounting for over 2.5 million new cases in 2022.^1^, ^2^ This growing burden poses economic and healthcare challenges, exacerbating global health disparities. Epigenetic alterations, particularly DNA methylation changes, play crucial roles in cancer initiation and progression, offering valuable opportunities for biomarker discovery and the development of therapies using hypomethylating agents.

Herein, we adopted an integrative approach to explore the DNA methylation landscape across the most common urological cancers (Figure S1). Genome‐wide DNA methylation profiling was performed using the Illumina HumanMethylation450 BeadChip on 72 fresh‐frozen tissue samples from a comprehensive cancer centre (GSE52955), including 25 PCa, 5 normal prostate, 14 BlCa, 5 normal bladder, 17 KCa, and 6 normal kidney (File S1 ‐ Table S1). Differentially methylated CpG sites were identified and integrated with The Cancer Genome Atlas (TCGA) methylation and RNA‐seq datasets to assess their impact on gene expression. Gene Ontology analysis was conducted to identify dysregulated pathways.

Unsupervised hierarchical clustering showed that DNA methylation patterns clearly distinguish tumours from normal tissues and further reflect their tissue of origin (Figure 1). Normal samples from prostate, bladder, and kidney clustered together, whereas tumours exhibited divergent profiles, especially PCa and BlCa, which showed more pronounced epigenetic reprogramming than KCa. These differences suggest tumour‐specific epigenetic divergence, possibly influenced by specific tumour microenvironment and cell‐type specialisation. Understanding these transitions from normal to malignant states may unveil biomarkers for screening and early detection, as well as therapeutic vulnerabilities.

**FIGURE 1:**
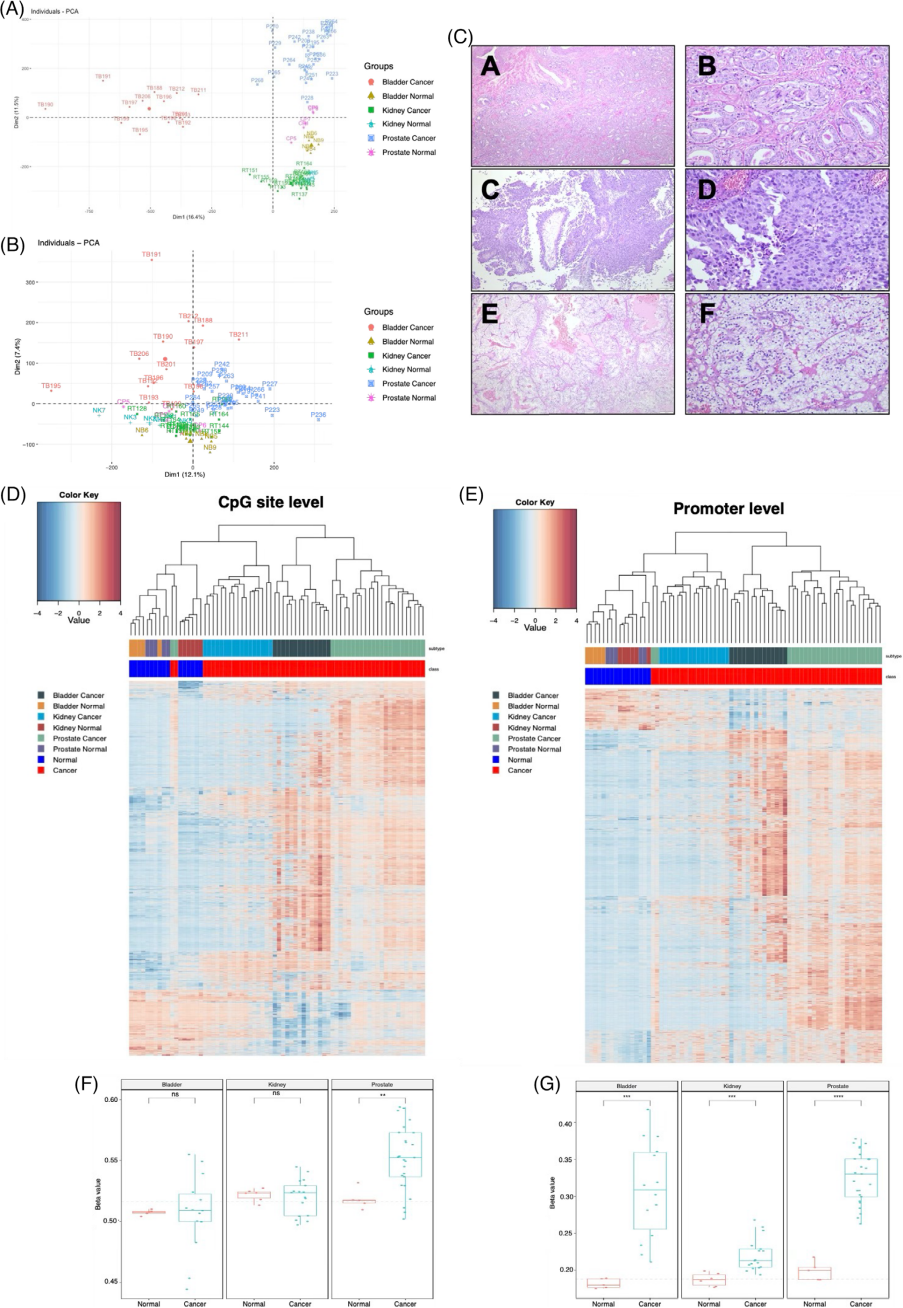
Principal component analysis (PCA), histological characterisation, and unsupervised clustering of urological tissue samples. (A, B) PCA plots based on individual CpG sites (A) and promoters (B), with samples colour‐coded according to their tissue of origin. (C) Representative histological images of the analysed samples (a and b – acinar adenocarcinoma of the prostate. Notice the perineural invasion in b. c and d – non‐invasive papillary urothelial carcinoma of the bladder, high‐grade. Notice the high‐grade features in d, with mitotic figures, irregular nuclei, and architectural disorganisation. e and f – clear cell carcinoma of the kidney, ISUP grade 2. a, c and e – 40× magnification; b, d and f – 200× magnification. (D, E) Unsupervised hierarchical clustering of samples based on methylation values at the CpG site level (D) and promoter level (E). (F, G) Mean methylation beta‐values comparing normal and cancerous tissues for each urological type, considering all CpG sites (F) and promoter CpG sites (G).

A key finding was the global hypomethylation observed in BlCa and KCa, contrasting with widespread hypermethylation in PCa (Figure 2, File S1 Table S1). Indeed, PCa disclosed predominant hypermethylation at both CpG sites (12 877) and promoters (2640), compared to only 2417 and 24 hypomethylated sites, respectively. In contrast, BlCa demonstrated extensive genome‐wide hypomethylation (40 788 hypomethylated vs. 15 961 hypermethylated CpG sites), although most promoter‐associated CpGs were hypermethylated (1882 hypermethylated vs. 436 hypomethylated). KCa exhibited fewer alterations overall, suggesting a more conserved epigenetic profile.

**FIGURE 2:**
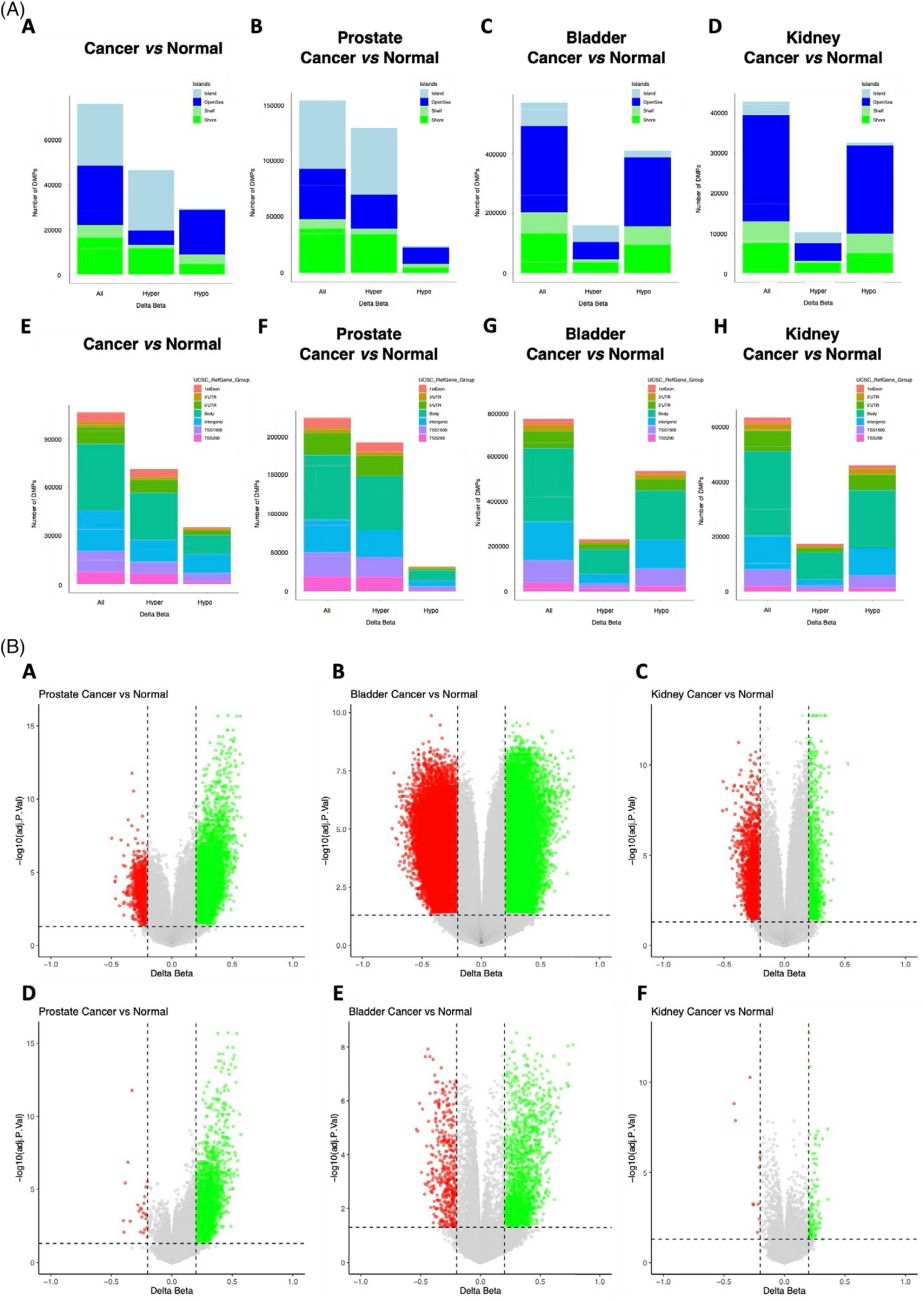
Differential methylation patterns across urological cancers. (A) Distribution of hyper‐ and hypomethylated CpG sites across comparisons. (a–d) Distribution of differentially methylated CpG sites (hyper‐ and hypomethylated) relative to CpG island locations for all cancer vs. normal samples (a), prostate cancer vs. normal prostate samples (b), bladder cancer vs. normal bladder samples (c), and kidney cancer vs. normal kidney samples (d). (e‐h) Distribution of differentially methylated CpG sites relative to gene locations for the same comparisons: all cancer vs. normal samples (e), prostate cancer vs. normal prostate samples (e), bladder cancer vs. normal bladder samples (g), and kidney cancer vs. normal kidney samples (h). (B) Volcano plots illustrating differential methylation across cancer and normal tissues for urological tumours. (a–c) Differential methylation at the CpG site level: (a) prostate cancer vs. normal prostate tissues, (b) bladder cancer vs. normal bladder tissues, and (c) kidney cancer vs. normal kidney tissues. (d–f) Differential methylation at the promoter level: (d) prostate cancer vs. normal prostate tissues, (e) bladder cancer vs. normal bladder tissues, and (f) kidney cancer vs. normal kidney tissues.

As global hypomethylation has been associated with genomic instability, increased mutational burden, and activation of oncogenic pathways,^3^, ^4^, ^5^ this may underlie BlCa and KCa's increased neoantigen load and consequent responsiveness to immune checkpoint inhibitors. These insights support the link between hypomethylation‐driven instability and immunotherapy sensitivity.

Conversely, PCa was hallmarked by promoter hypermethylation, suggesting a tumour biology driven by silencing of tumour suppressor genes. This may also have therapeutic implications, as hypomethylating agents (already approved for haematological malignancies) may be particularly effective in counteracting PCa progression.^6^ Also, it may facilitate the development of biomarker panels of hypermethylated gene promoters for PCa patients' diagnosis and monitoring, including through liquid biopsy testing.

Venn diagram analysis revealed both shared and cancer‐specific methylation signatures (Figure 3). PCa and BlCa shared 1176 hypomethylated and 2816 hypermethylated CpG sites, including 538 hypermethylated sites in promoter regions, suggesting convergent epigenetic mechanisms. Although these common features may enable the development of pan‐cancer biomarkers for early detection, the predominance of cancer‐specific patterns underscores the importance of context in biomarker development and therapy design.

**FIGURE 3:**
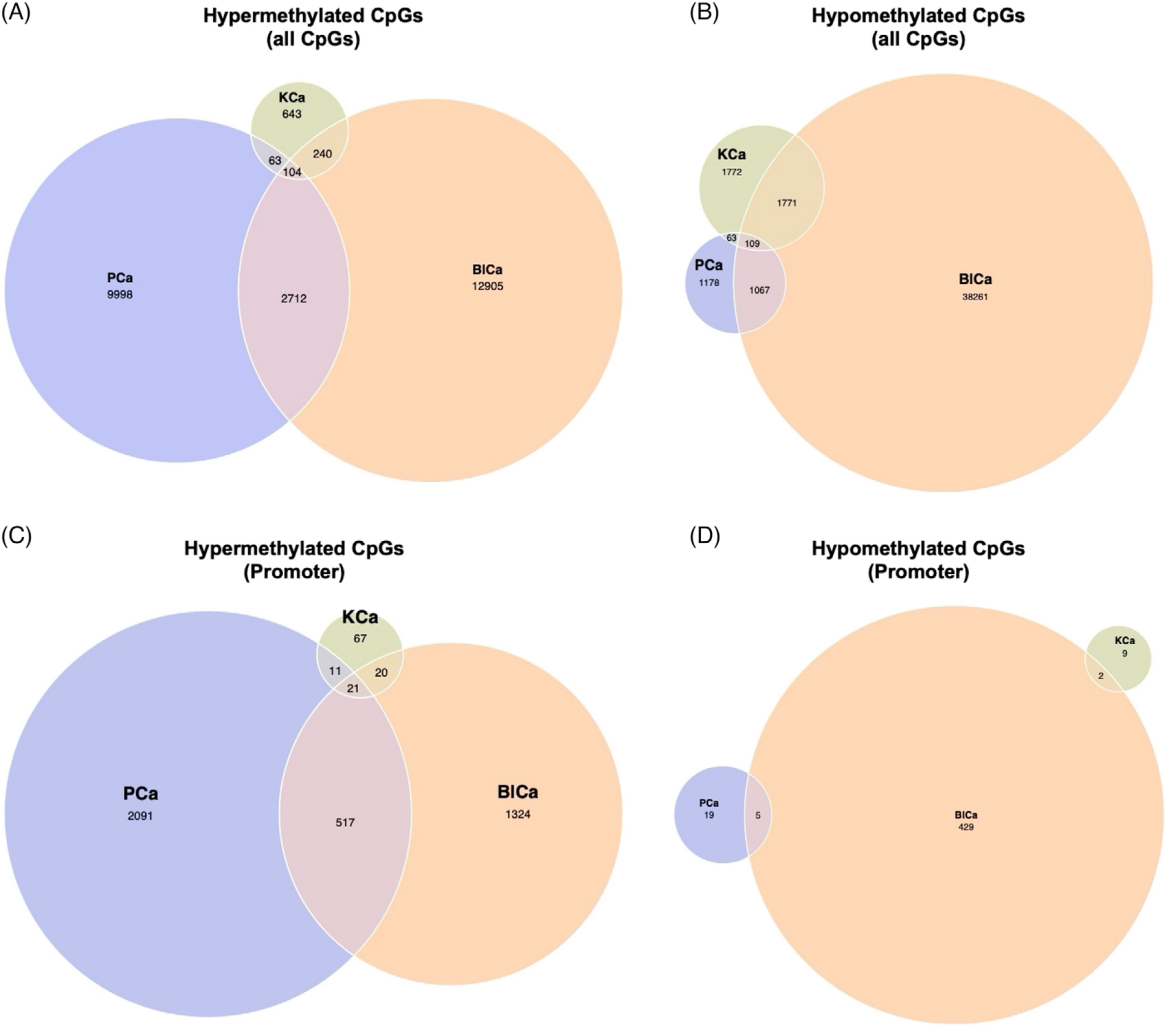
Venn diagram analysis of hypermethylated and hypomethylated CpG sites across urological cancers. (A, C) Common and unique hypermethylated CpG sites in prostate, bladder, and kidney cancers at the genome‐wide level (A) and within CpG islands (C). (B, D) Common and unique hypomethylated CpG sites in prostate, bladder, and kidney cancers at the genome‐wide level (B) and within CpG islands (D).

Integration with TCGA datasets showed that hypermethylated CpGs in PCa and BlCa were associated with silencing of eight key tumour suppressors and regulators (File S2), involved in DNA repair (*DMC1*, *ZNF154*), oxidative stress (*CDO1*, *BHMT2*), epithelial‐mesenchymal transition (*HOXA7*, *MDFI*), immune evasion (*NKAPL*, *ZNF154*), and oncogenic signalling (*RSPH9*). This suggests both shared and cancer‐specific tumourigenic pathways that may be epigenetically regulated.

In PCa, promoter hypermethylation predominantly downregulated genes involved in metabolism, detoxification, and molecular transport (Figure 4A, File S2), including *GSTP1*, *GSTM2*, *GPX3*, and *GPX7*.^7^ Impaired metabolic and detoxification pathways may lead to the accumulation of mutagenic intermediates, driven by chronic oxidative stress.^8^ Furthermore, epigenetic repression of solute carrier transporters may reduce amino acid bioavailability, disrupting metabolic rewiring, redox balance, and nutrient homeostasis, and impacting tumour therapy response.^9^ Targeting these dysregulated pathways with epigenetic drugs may offer novel therapeutic strategies in PCa.

**FIGURE 4:**
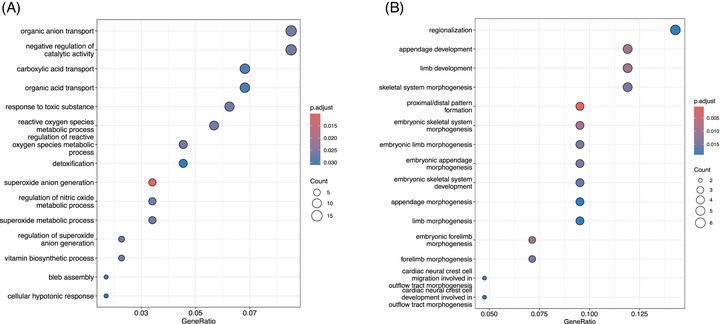
Top 15 enriched Gene Ontology (GO) terms associated with hypermethylated genes that exhibit a significant negative correlation with gene expression in urological cancers. (A) Prostate cancer. (BA) Bladder cancer.

In BlCa, promoter hypermethylation contributed to silence genes implicated in cell fate, tissue organisation, and structural integrity (Figure 4B, File S1). Loss of these programs often result in stem‐like phenotypes, altered cell adhesion, ECM remodeling, and EMT dysregulation. Importantly, several HOX family genes (including *HOXA11*, *HOXA11AS*, *HOXA9*, *HOXC10* and *HOXC9*) were notably affected. These genes encode transcription factors and non‐coding RNAs essential for embryonic development, cellular physiology, and tissue homeostasis, further implicating epigenetic dysregulation in BlCa progression.^10^

Overall, the integration of our dataset with TCGA emphasises the translational relevance of methylation patterns identified in these urological cancers, reinforcing their role in tumourigenesis. It further supports the exploitation of DNA methyltransferase (DNMT) inhibitors in urological cancers treatment, although, the therapeutic implications may differ across cancer types given their distinct epigenetic landscapes. In PCa, where widespread promoter hypermethylation predominates, DNMT inhibitors are expected to exert greater efficacy by reactivating silenced tumour suppressor genes. In contrast, BlCa and KCa, which display global hypomethylation, are likely less responsive to DNMT inhibition as monotherapies. Nonetheless, focal promoter hypermethylation events, specific molecular subgroups, and combination strategies (e.g., with immunotherapy or targeted therapies) may render subsets of these tumours sensitive to DNMT inhibition. This underscores the importance of biomarker‐driven patient stratification and combinatorial approaches to fully exploit the therapeutic potential of DNMT inhibitors in these cancers. Preferentially hypermethylated pathways, such as those identified in PCa and BlCa, represent promising targets for combined epigenetic and targeted therapies. Ultimately, the success of these approaches will depend on the development of reliable biomarkers to guide treatment selection and monitor therapeutic responses, further highlighting the importance of precision medicine.

In conclusion, our findings provide a cross‐cancer comparison of epigenetic regulation among major urological malignancies. Whereas some shared mechanisms were depicted, distinct cancer‐type‐specific methylation patterns emerged, reflecting the complexity and heterogeneity of tumour epigenomes. These findings not only deepen our understanding of urological cancer biology but also support the development of methylation‐based biomarkers for early detection and tailored therapeutic interventions that leverage tumour‐type‐specific epigenetic vulnerabilities. With the growing availability of circulating DNA methylation assays and the emergence of epigenetic therapies, we consider these findings timely and clinically relevant.

## AUTHOR CONTRIBUTIONS

CJ, RH, VC, JL, JRC, and ME conceived and designed the experiments. JRC performed the molecular experiments. VC and AG performed bioinformatic analyses. VC and JL analysed the data. JL performed pathological revision. IC processed clinical samples. RF provided patients’ clinical information. VC and JL drafted the manuscript. CJ and RH supervised the work and revised the manuscript. All authors read and approved the manuscript.

## CONFLICT OF INTEREST STATEMENT

The authors declare that they have no competing interests.

## FUNDING INFORMATION

This work was funded by IPO Porto Research Center (Grant PI 27‐CI‐IPOP‐27‐2016) and national funds through FCT – Fundação para a Ciência e Tecnologia, within the scope of the project 10.54499/2022.05135.PTDC. VC was supported by a fellowship from the ‘la Caixa’ Foundation (ID 100010434). The fellowship code is LCF/BQ/DR20/11790013.

## ETHICS STATEMENT

This study was approved by the Ethics Committee (CES‐IPOPFG‐EPE 205/2013) of the Portuguese Oncology Institute of Porto, Portugal. All procedures performed in tasks involving human participants were under the ethical standards of the institutional and/or national research committee and with the 1964 Helsinki declaration and its later amendments or comparable ethical standards.

## Supporting information


**Figure S1**. Workflow illustrating the DNA methylation profiling of in‐house fresh‐frozen urological cancer tissues using the Illumina HumanMethylation450 BeadChip, and the integrative analysis with The Cancer Genome Atlas (TCGA) datasets. Differentially methylated CpG sites were identified and correlated with gene expression changes to reveal functionally relevant alterations and affected biological pathways. Created in BioRender.com.

**File S1**. Supplementary materials.

**File S2**. Excel containing shared and cancer‐specific hypermethylated and hypomethylated genes. Only genes with a significant CpG methylation and gene expression correlation of *R* < –.3 and at least two CpGs showing this correlation were included.

## Data Availability

The data that support the findings of this study are openly available in in the GEO repository under accession number GSE52955.
