# Supplementary material for: Large‐scale DNA methylation profiling of urological cancers identifies shared and cancer‐specific methylation signatures, and reveals differentially activated pathways for therapeutic targeting
Source: Clin Transl Med. 2025 Oct 13;15(10):e70488. doi: 10.1002/ctm2.70488 (PMC12518500; doi:10.1002/ctm2.70488)
Supplement: Supplementary file 1 — Figure S1. Workflow illustrating the DNA methylation profiling of in‐house fresh‐frozen urological cancer tissues using the Illumina HumanMethylation450 BeadChip, and the integrative analysis with The Cancer Genome Atlas (TCGA) datasets. Differentially methylated CpG sites were identified and correlated with gene expression changes to reveal functionally relevant alterations and affected biological pathways. Created in BioRender.com. [file CTM2-15-e70488-s002.pdf]

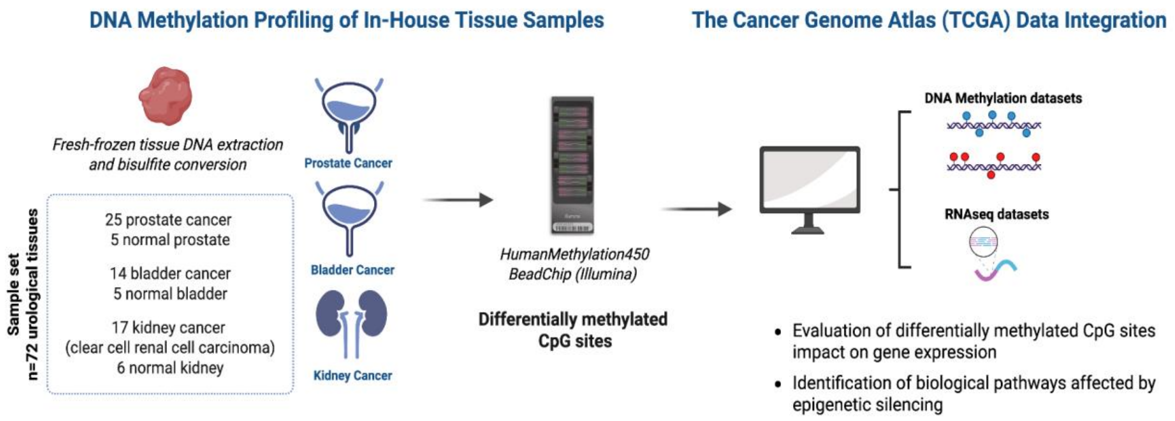
DNA Methylation Profiling of In-House Tissue Samples
The Cancer Genome Atlas (TCGA) Data Integration
Fresh-frozen tissue DNA extraction and bisulfite conversion
Sample set n=72 urological tissues
25 prostate cancer
5 normal prostate
14 bladder cancer
5 normal bladder
17 kidney cancer (clear cell renal cell carcinoma)
6 normal kidney
Prostate Cancer
Bladder Cancer
Kidney Cancer
HumanMethylation450 BeadChip (Illumina)
Differentially methylated CpG sites
DNA Methylation datasets
RNAseq datasets
Evaluation of differentially methylated CpG sites impact on gene expression
Identification of biological pathways affected by epigenetic silencing
