# Supplementary material for: Large‐scale DNA methylation profiling of urological cancers identifies shared and cancer‐specific methylation signatures, and reveals differentially activated pathways for therapeutic targeting
Source: Clin Transl Med. 2025 Oct 13;15(10):e70488. doi: 10.1002/ctm2.70488 (PMC12518500; doi:10.1002/ctm2.70488)
Supplement: Supplementary file 2 — File S1. Supplementary materials. [file CTM2-15-e70488-s003.docx]

**Supplementary Materials**

**Material and Methods**

*Clinical Samples*

Thirty prostate tissues (25 PCa and 5 normal from cystoprostatectomy patients with urothelial neoplasms and no prostate cancer or high-grade prostatic intraepithelial neoplasia), 19 bladder (14 BlCa and 5 normal from patients undergoing cystectomy or transurethral resections without evidence of urothelial neoplasms) and 23 kidney (17 KCa – all clear cell RCC – and 6 normal kidney tissues from patients without renal cell neoplasms) tissues were selected. Clinicopathological characterization is depicted in Table S1. All cases were reviewed by a Uropathology-dedicated pathologist, (re)classified according to the most recent 2022 WHO Classification and staged according to American Joint Committee on Cancer staging system, 8^th^ Edition. All patients were treated by the same multidisciplinary team at the Urology Clinic of Portuguese Oncology Institute of Porto, Portugal. Clinical information was obtained from patients’ charts. This study was approved by the institutional review board of Portuguese Oncology Institute of Porto, Portugal (CES-IPOPFG-EPE205/2013).

*DNA extraction, Bisulfite conversion and Methylation Analysis*

Freshly collected tissues were snap-frozen in isopentane solution and stored at -80ºC until further processing. After histological confirmation, tissue fragments were trimmed to achieve >70% target cell content. To minimize the potential for genetic heterogeneity from sampling multiple clones, DNA was extracted from histopathologically verified tissue sections.

DNA was extracted using the phenol-chloroform method (Sigma) and 1000 ng of genomic DNA was bisulfite converted using EZ DNA Methylation Kit (Zymo Research), following the manufacturer’s instructions. All DNA samples were assessed for integrity, quantity, and purity by electrophoresis in a 1.3% agarose gel, picogreen quantification, and nanodrop measurements. All samples were randomly distributed into 96-well plates. Converted DNA (200ng) hybridized on the HumanMethylation450 BeadChip (Illumina), assessing approximately 450,000 CpG sites.

*Bioinformatic and Statistical Analyses*

1. HumanMethylation450 BeadChip data: IPOPorto cohort

HumanMethylation450 BeadChip data were processed using the Bioconductor “minfi” package (version 1.44) in R statistical software (version 4.2.3). Data normalization was performed using the “Illumina” procedure, which replicates GenomeStudio’s (Illumina) approach, including background correction and normalization with the first array on each plate as a reference.

Probes containing single nucleotide polymorphisms (SNPs) with a minor allele frequency (MAF) >1 % (1000 Genomes) in the first 10 bp of the interrogated CpG site were excluded. Methylation levels (β-values) for each of the 485,577 CpG sites were calculated as the ratio of methylated signal divided by the sum of methylated and unmethylated signals, multiplied by 100. β values range from 0 (no methylation) to 1 (100% methylation of both alleles). After normalization, probes associated with the X and Y chromosomes were removed. All analyses were conducted using the human genome reference version 19 (hg19). Processed data were deposited in the GEO repository under accession number GSE52955.

Differential methylation analyses were performed using linear regression models, considering sex and age as covariates. Differentially methylated probes were considered significant if they had an adjusted p-value (False Discovery Rate, FDR) < 0.05. Probes with a β-value increase > 0.2 in cancer samples relative to normal samples were classified as “hypermethylated”, whereas probes with a β-value decrease <-0.2 were classified as “hypomethylated”.

Boxplots were used to visualize differences in methylation levels between samples groups (e.g., Normal vs. Cancer), with significance assessed via the Wilcoxon rank-sum test. Statistical significance was denoted by stars: 4 stars p-value <0.0001, 3 stars p-value < 0.001, 2 stars p-value < 0.01, and 1 star p-value < 0.05.

Volcano plots were employed to illustrate statistical significance (adjusted p-value) against the magnitude of methylation change (Delta Beta, defined as the difference in β- values between 2 groups of samples). CpGs with adjusted p-value <0.05 and Δβ>0.2 (hypermethylated) or Δβ<-0.2 (hypomethylated) were deemed biologically significant. On volcano plots, hypermethylated CpGs are positioned towards the right, hypomethylated towards the left, and the most statistically significant CpGs towards the top.

2. *In silico* analyses: The Cancer Genome Atlas (TCGA) cohort

To validate our findings, we conducted an *in-silico* analysis using The Cancer Genome Atlas (TCGA) dataset, including TCGA-PRAD (n=497) for PCa and TCGA-BLCA (n=407) for BlCa. Clinical data, RNA-seq expression data, and DNA methylation (450k) data were retrieved via the UCSC Xena browser (22) and analyzed using R (version 4.4.1, MacOS).

Our stepwise analysis included: (1) Identifying and validating differentially methylated CpG sites in BlCa and PCa; (2) Assessing the functional impact of these alterations (shared and cancer-specific hyper- or hypomethylated CpG sites in promoter regions) by correlating DNA methylation with gene expression. Differential methylation analysis between tumor and normal samples was performed using the Wilcoxon rank-sum test, with p-values adjusted for multiple testing via the Benjamini-Hochberg method. CpG sites with adjusted p-values <0.05 were considered significantly differentially methylated and selected for further analysis. To explore the relationship between DNA methylation and gene expression, Pearson correlation analyses were conducted for each CpG-gene pair using matched tumor samples. We focused on negative correlations, indicating inverse relationships between methylation and expression, and considered correlation significant when p<0.05 and R <-0.3. To ensure robustness, we further filtered for genes with multiple significantly correlated CpG sites.

Gene Ontology (GO) enrichment analysis of the selected genes was conducted using the clusterProfiler package in R. The enrichGO function was applied with multiple testing correction via the Benjamini-Hochberg method. The top 15 significantly enriched terms were visualized in a dot plot to investigate the biological significance of the validated genes in urological cancers.

**Table S1 –** **Clinicopathological characterization of the study cohort.**

| **Clinicopathological features** | **N (%)** |
| --- | --- |
| **PROSTATE CANCER (N=25)** | |
| Age (median, IQR) | 66 (61-68) |
| PSA (median, IQR) | 8.8 (5.4-12.3) |
| WHO Gleason Groups |  |
| Group 1 (G 3+3) | 10/25 (40) |
| Group 2 (G 3+4) | 11/25 (44) |
| Group 3 (G 4+3) | 3/25 (12) |
| Group >=4 | 1/25 (4) |
| pT Stage |  |
| pT2 | 13/25 (52) |
| pT3a | 9/25 (36) |
| pT3b | 3/25 (12) |
| Disease recurrence |  |
| No | 13/25 (52) |
| Yes | 9/25 (36) |
| No remission | 3/25 (12) |
| Death from disease |  |
| No | 23/25 (92) |
| Yes | 2/25 (8) |
| **NORMAL PROSTATE (N=5)** | |
| Age (median, range) | 61(49-80) |
| **BLADDER CANCER (N=14)** | |
| Age (median, range) | 70 (62-77) |
| Gender |  |
| Female | 3/14 (21) |
| Male | 11/14 (79) |
| Histological subtype |  |
| Papillary urothelial carcinoma, low-grade | 8/14 (57) |
| Papillary urothelial carcinoma, high-grade | 4/14 (29) |
| Invasive urothelial carcinoma, high-grade | 2/14 (14) |
| Muscle invasion |  |
| NMIBC | 12/14 (86) |
| MIBC | 2 (14) |
| pT Stage |  |
| pTa | 6/14 (43) |
| pT1 | 6/14 (43) |
| pT2 | 1/14 (7) |
| pT4 | 1/14 (7) |
| Disease recurrence |  |
| No | 6/14 (43) |
| Yes | 8/14 (57) |
| Death from disease |  |
| No | 12/14 (86) |
| Yes | 2/14 (14) |
| **NORMAL BLADDER (N=5)** | |
| Age (median, range) | 61 (55-75) |
| Gender |  |
| Female | 0/5 (0) |
| Male | 5/5 (100) |
| **KIDNEY CANCER (N=17)** | |
| Age (median, range) | 57 (51-66) |
| Gender |  |
| Male | 11/17 (65) |
| Female | 6/17 (35) |
| pT Stage |  |
| pT1 | 12/17 (70) |
| pT2 | 2/17 (12) |
| pT3 | 3/17 (18) |
| Stage |  |
| I | 12/17 (70) |
| II | 2/17 (12) |
| III | 2/17 (12) |
| IV | 1/17 (6) |
| ISUP Grade |  |
| 2 | 10/17 (59) |
| 3 | 7/17 (41) |
| Death from disease |  |
| No | 16/17 (94) |
| Yes | 1/17 (6) |
| **NORMAL KIDNEY (N=6)** | |
| Age (median, range) | 55 (39-77) |
| Gender |  |
| Female | 1/6 |
| Male | 5/6 |

Abbreviations: MIBC – muscle invasive bladder cancer; NMIBC – non-muscle invasive bladder cancer.

**Table S2 –** **Number differentially hypermethylated and hypomethylated CpG sites genome-wide and within CpG islands for each urological cancer type.**

|  | CpG sites (genome-wide)  (hyper-/hypomethylated) | CpG sites (Islands) (hyper-/hypomethylated) |
| --- | --- | --- |
| Prostate Cancer *vs* Normal Prostate | 12877/2417 | 2640/24 |
| Bladder Cancer *vs* Normal Bladder | 15961/40788 | 1882/436 |
| Kidney Cancer *vs* Normal Kidney | 1050/3294 | 119/11 |
